# Inadequate representation of individuals of African ancestry in pharmacogenetics of tamoxifen research

**DOI:** 10.1111/cts.70029

**Published:** 2024-09-19

**Authors:** Keneuoe Cecilia Nthontho, Andrew Khulekani Ndlovu, Giacomo Maria Paganotti

**Affiliations:** ^1^ School of Allied Health Professions, Faculty of Health Sciences University of Botswana Gaborone Botswana; ^2^ Botswana‐University of Pennsylvania Partnership Gaborone Botswana; ^3^ Division of Infectious Diseases, Perelman School of Medicine University of Pennsylvania Philadelphia Pennsylvania USA

Dear Editor,

We read with great interest the paper titled “Pharmacogenetics of tamoxifen in breast cancer patients of African descent: Lack of data” by Kruger et al.^1^ We agree with the authors, on their analysis and conclusions from their work. Indeed, no studies in Africans or in populations of African ancestry have looked and successfully demonstrated any association between inherited variants in genes underlying tamoxifen metabolism and clinical outcomes. Actually, only a very limited number of studies both among Africans and/or subjects of African descent explored the association between genetic variants of *CYP2D6* and tamoxifen/endoxifen concentration, association that has been proved and confirmed.^2^ Another level of complexity is that *CYP2D6* “star alleles” influence tamoxifen/endoxifen level concentrations, but a significant number of African polymorphisms have not been fully explored and validated. A point of interest and possible evaluation could also be that whereas women of African descent in the West may be an appropriate proxy for sub‐Saharan African populations, the disease landscape of women in sub‐Saharan Africa may differ from those from other continents. In fact, it should be taken into consideration the standing co‐infections with parasitic, bacterial and viral diseases, and their treatments, together with malnutrition. This may complicate the general picture and the interactions between drugs, diseases and pharmacogenetics of transport and metabolism. Additionally, there are numerous studies assessing the role of the genes encoding for enzymes relevant for tamoxifen metabolism, but the present literature points in the context of antimalarial and antiretroviral drugs.^3^ These findings may provide very useful information for allele frequencies that can be translated to infer tamoxifen metabolism.

Here, we want to stress that *lack of data* should be addressed with appropriate clinical studies in subjects from African countries and among different African ethnic groups, as the available dosing guidelines are likely to be inappropriate for most Africans.^4^ Nowadays, “omics” technologies and novel approaches allow for a deep understanding of these phenomena, also in the context of Africa, and this constitute a mandatory goal for scientists in the area.

Also, it is important to note that with all the concepts that have been raised, there is still the issue of *lack of funding* for research in pharmacogenetics for the continent, as well as initiatives to set up organizations that could best aid in providing the necessary visibility and resources to shape up the story of pharmacogenetics in Africans and populations of African ancestry.

## FUNDING INFORMATION

No funding was received for this work.

## CONFLICT OF INTEREST STATEMENT

The authors declared no competing interests for this work.

## Data Availability

Inquiries about data availability should be directed to the authors.
